# Risk stratification during antenatal care failed to identify most mothers who experienced adverse pregnancy outcomes: A prospective study from Kakamega County, Kenya

**DOI:** 10.1111/tmi.14110

**Published:** 2025-04-06

**Authors:** Jan E. Cooper, Margaret Kruk, David Kapaon, Kennedy Opondo, Jacinta Nzinga, Rose J. Kosgei, Kevin Croke

**Affiliations:** ^1^ Department of Global Health and Population Harvard T.H. Chan School of Public Health Boston Massachusetts USA; ^2^ Department of Medicine, Division of General Medicine and Geriatrics Washington University School of Medicine USA; ^3^ Department of Epidemiology Boston University School of Public Health USA; ^4^ Health Economics Research Unit, Kenya Medical Research Institute‐Wellcome Trust Research Programme, Department of International Public Health Liverpool School of Tropical Medicine UK; ^5^ Department of Obstetrics and Gynecology University of Nairobi Kenya

**Keywords:** adverse delivery outcomes, health systems, pregnancy risk

## Abstract

**Introduction:**

Risk stratification of pregnancies informs clinical care globally. Yet recent research has cast doubt on the ability of currently used population‐level risk measures to accurately predict poor outcomes at the individual level. We examine the assumption that existing forms of risk stratification can successfully identify women likely to develop complications during delivery in a rural setting in Kenya.

**Methods:**

We conducted a prospective observational study of 19,653 pregnant women in Kakamega County in Western Kenya. Women were contacted three times during the perinatal period and surveyed about provider‐identified risks and self‐assessed concerns about pregnancy complications, delivery process outcomes, and adverse delivery outcomes. Measures of risk were derived from women's self‐reporting. We compared delivery process outcomes and adverse delivery outcomes between high‐ and low‐risk pregnancies, and between women with and without expressed concerns about delivery complications. Delivery process outcomes included intrapartum referral, unplanned caesarean section, blood transfusion, hysterectomy, or admission to an intensive care unit. Adverse delivery outcomes included stillbirth, neonatal mortality, and maternal mortality. We reported means and confidence intervals for each category, and tested for differences using bivariate linear regression.

**Results:**

Thirty‐eight percent of pregnancies had at least one risk factor consistent with a high risk pregnancy; the remaining 62% were low risk by this criteria. Rates of most adverse process outcomes and delivery outcomes were higher among pregnancies with known risks. However, 64.5% of maternal deaths and 54.8% of all deaths in the sample took place among pregnancies characterised as low risk.

**Conclusions:**

Risk stratification using existing indicators of risk during pregnancy is inadequate to identify women at risk of adverse health outcomes in this setting.

## INTRODUCTION

Globally, classifying pregnant women based on their risk of delivery complications is a key aspect of antenatal and maternal care. In low‐income settings, access to hospitals equipped to provide comprehensive, definitive care in case of emergencies is often limited. As a result, when facing decisions about how to allocate limited resources, policymakers have often prioritised pregnant women at highest risk of adverse health outcomes by identifying them during antenatal care and referring them for hospital‐based delivery care.

Yet while this form of risk‐stratification is widely practiced, its efficacy is poorly understood. It assumes that the factors which are correlated with adverse outcomes at the population level can accurately predict risk for individual women. In practice, this would require pregnancy risk measures to be very sensitive tests, with few false negatives: while false positives (women with low‐risk pregnancies advised to give birth in hospitals) might be largely benign, false negatives (in which high‐risk women with unidentified high‐risk pregnancies are advised to delivery in primary‐level clinics) can be dangerous if those clinics are not equipped to provide appropriate care when complications arise. This is particularly hazardous in regions where referral systems are weak and where primary care clinics are far from higher‐level facilities—common characteristics of settings with high maternal and newborn mortality.

Despite the widespread de facto reliance on risk‐stratification in low‐ and middle‐income countries, particularly in regions with high maternal and newborn mortality, the model merits examination, especially in light of recent research that has cast doubt on the ability of existing measures of risk to accurately predict poor individual outcomes across a range of contexts [1, 2, 3, 4]. Even in settings with close to universal uptake of early ANC, and which include many diagnostic tests, the result of which are captured in rich administrative data or electronic health records, such risk stratification has failed to predict a substantial proportion of complications [5]. The situation is likely more challenging in low‐ and middle‐income settings where antenatal care coverage is lower, occurs later in pregnancy, and includes fewer diagnostic tests and lower accuracy of clinical diagnosis.

This re‐evaluation is warranted given current efforts to reduce maternal and newborn mortality in low‐ and middle‐income countries. While the past two decades have seen notable declines in maternal and newborn mortality rates, more recently this progress has stagnated, highlighting the need for high‐quality care throughout the perinatal period. In response, many high‐mortality settings are re‐evaluating their maternal and newborn care models to address persistent challenges related to system capacity, accessibility, and quality of care. Kenya, where neonatal mortality is currently 21/1000 births, is illustrative of these efforts [6]. The Kenyan government has implemented a number of policies to improve access to maternal and newborn care, including the removal of user fees as well as the creation of an insurance programme to ensure free delivery care. Yet despite these efforts, many pregnant women still face barriers accessing high‐quality care throughout their pregnancy, childbirth, and postnatal period. Additionally, the country faces a shortage of fully equipped hospitals with specialised facilities, such as operating theatres and newborn units, and has both an absolute shortage and inequitable distribution of specialised clinicians, especially in rural areas. As such, the identification of mothers at high risk of adverse outcomes is a priority, since they might otherwise face significant obstacles in accessing appropriate care.

Kenya's recently revised obstetric and perinatal care guidelines reflect national policies and practice regarding risk assessment during pregnancy. The country's previous guidelines, from 2011, noted that “life threatening complications of pregnancy are difficult to predict with any degree of certainty. Health care providers must, therefore, consider the possibility of complications in every pregnancy and prepare clients accordingly. While risk assessment can help direct counselling and treatment for individuals, it is important to understand that most women who experience complications have no ‘risk factors’ at all.” [7] However, Kenya's recently updated guidelines make clear that existing policy still relies on a risk assessment which places significant weight on antenatal care's ability to determine risk [8]. Specifically, Kenya's 2024 National Guidelines on Quality Obstetrics and Perinatal Care state that women with low risk pregnancies  should choose “any birth setting” with a skilled attendant present, and notes that providers should “advise low risk nulliparous women that they should have emergency preparedness as medical or obstetric complications can arise necessitating referral.” (p. 113). For high risk pregnancies, the guidelines indicate that birth should take place in a health facility.

Using data from a rural setting in western Kenya (Kakamega County), we examine the assumption that risk stratification during antenatal care can successfully identify a large percentage of at‐risk pregnancies. This analysis can shed light on whether risk stratification using existing tools and measures is an effective strategy to target needed care in settings with high rates of maternal and newborn mortality.

## METHODS

### Data collection

This study uses data from respondents who accessed antenatal care (ANC) in 72 facilities sampled from all 253 public facilities providing ANC across the 12 sub‐counties in Kakamega. These enrollment sites were selected using a two‐stage stratified sampling proportional to size, where the probability of selection was based on the facility population of first ANC visits. Privately owned health services, clinics in Kakamega town, and low volume facilities were all excluded. Data collection began in February 2022, and follow‐up was conducted for all births occurring up to March 2023. Further details of the sampling process are described elsewhere [9].

At the start of enrollment in February 2022, all women presenting for ANC were invited to participate in the study. Subsequently, women were recruited into the sample when they first accessed ANC. Pregnant women were eligible to enrol in the study if they were over 16 years old and reported plans to give birth in Kakamega County. All participants signed an informed consent form before enrolling in the study, which detailed the frequency of contacts and that participants would be asked about their health and childbirth experiences. Respondents who reported firm plans to give birth outside of Kakamega, or who were under 16 years old, were excluded from the sample. Enrolled women were contacted three times throughout the perinatal period: First, during enrollment at their ANC visit; second, by phone starting from 7 days after giving birth and third, by phone from 1 month after birth [4]. Any participants who could not be reached by phone were visited by an enumerator in person. At enrollment, women were asked about their health status, birth history, intended delivery facility, and health history of the current pregnancy, including any pregnancy‐related conditions or risk factors that they had been informed of by a provider. During the second contact, women were asked about intrapartum referrals, where they gave birth, birth complications, birth outcomes, and their length of stay at the facility after birth. One month after birth, women were again asked about delivery process outcomes, delivery outcomes, and postnatal care use. Mortality events were either reported by the mother, another family member, or a close contact who responded to follow‐up phone calls.

### Variable construction

We define pregnancy risk based on women's self‐reported history prior to their current pregnancy and reported conditions during this pregnancy. Following previous literature on markers of pregnancy risk [2], women were classified as having a high‐risk pregnancy if they experienced a previous neonatal death, previously gave birth via c‐section, were over 35 years old or under 20 years old, or had been told by a provider they had any one of the following conditions: chronic high blood pressure, chronic diabetes, gestational hypertension, gestational diabetes, anaemia, or placenta previa during their current pregnancy, or if they were carrying multiples. These indicators reflected risk factors most likely to be identified by the health system in Kenya using the current standard of ANC. We also examined a second definition of risk based on the respondent's self‐reported level of concern about experiencing pregnancy complications. During the enrollment survey, participants were asked: “How concerned are you that you will have complications during this pregnancy?” A binary classification of self‐reported concerns was coded as follows: women who responded as somewhat, moderately, or extremely concerned about complications during pregnancy were classified as having concerns, while women reporting they were slightly, or not at all concerned were classified as having no concerns about pregnancy complications. The correlation between having received any clinical diagnosis indicating a high‐risk pregnancy and women's subjective concerns about pregnancy complications was 0.038.

We examine two outcome categories: delivery process outcomes and adverse obstetric outcomes. Delivery process outcomes are were defined as equal to 1 for women who experienced intrapartum referral before reaching their final delivery facility, experienced an unplanned C‐section, received a blood transfusion, had a hysterectomy, or were admitted to an intensive care unit (ICU). These self‐reported outcomes were selected as measures of clinical intervention most likely to correlate with having experienced a maternal ‘near‐miss’ event [10]. Adverse obstetric outcomes were defined as equal to 1 if the mother or child experienced a stillbirth, neonatal mortality, or maternal mortality. The analysis was conducted using each pregnancy (rather than each birth) as the unit of analysis. Therefore, in cases of multiple pregnancy, respondents were defined as having experienced an adverse outcome if the mother or any one of the multiple newborns experienced an adverse outcome. A composite mortality outcome was also included, defined as the occurrence of any one of the included mortality events: stillbirth, neonatal mortality, or maternal mortality.

To examine the relationship between risk assessment and adverse outcomes, we compared delivery process outcomes between women whose pregnancies were classified as high‐risk for complications relative to women with pregnancies classed as low‐risk for complications. In addition, we examined the relationship between women's self‐reported concerns of complications and adverse delivery process outcomes. We also analysed rates of adverse obstetric outcomes between women whose pregnancies were high or low‐risk, and among women reporting concerns about pregnancy complications compared to women without concerns. Summary statistics were calculated using analytic weights that represent each respondent's probability of selection in the sample. We report means and confidence intervals for each category and test for differences in means using bivariate linear regressions with risk status as the sole independent variable, clustering standard errors by enrollment facility.

## RESULTS

A total of 19,653 respondents were included in the analysis, of whom 19,118 (96.8%) lived in Kakamega County. The mean age of women in the sample was 25.2 years, and 6725 (32.3%) had secondary education or higher. On average, respondents were in their sixth month of pregnancy at the time of enrollment, and 6624 (33.4%) were in their first pregnancy. Among women in the sample, 7612 (37.9%) were classified as having high‐risk pregnancies according to the definition provided in the previous section. 2482 (13.1%) of women in the sample reported feeling somewhat, moderately, or extremely concerned about pregnancy complications (Table 1).

**TABLE 1:** Participant characteristics.

Observations	*N* = 19,653
Respondent age^a^	25.2 (6.0)
Secondary or higher education	6,725 (32.4%)
Lives in Kakamega county	19,118 (96.8%)
Months of pregnancy at time of enrollment^a^	6.1 (1.8)
First pregnancy	6624 (33.4%)
High‐risk pregnancy	7612 (37.9%)
Somewhat/moderately/extremely concerned about complications	2482 (13.1%)

^a^ Continuous variables presented as: mean (SD); binary variables presented as: *n* (%). High risk pregnancy defined as previous neonatal death; mother over 35 years old or under 20 years old; or diagnosed with high blood pressure, gestational diabetes, placenta previa.

**FIGURE 1:**
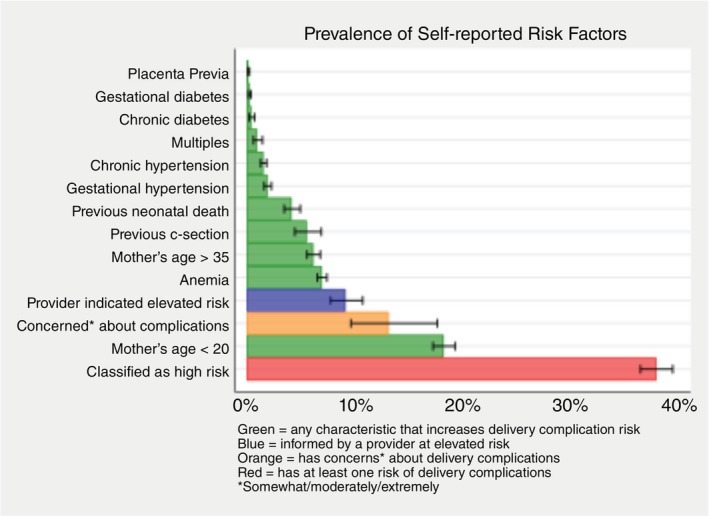
Mothers' self‐report of risk factors. Proportion of respondents with: (i) each risk factor; (ii) those who have been informed by a provider that they are at elevated risk, (iii) any characteristics that increase the risk of delivery complications, or (iv) indicate that they have concerns about delivery complications.

Among women in the sample, 2230 (11.3%) had been informed by their provider that their pregnancy had at least one specific medical condition that was a risk factor for complications. Of these, the most common condition was anaemia, reported by 1502 women (7.7% of the sample), followed by gestational hypertension *n* = 408 (2.1%), carrying multiples *n* = 175 (0.9%), gestational diabetes *n* = 49 (0.3%), and placenta previa *n* = 44 (0.2%). Additional risk factors were classified based on information about mothers' demographic status and reproductive history, as well as other relevant chronic health conditions. The most common of these pregnancy risk factors were young maternal age (under 20 years old), reported by 3390 women (17.3% of the sample), and advanced maternal age (over 35 years old) reported by 1372 women (7.0% of the sample). Other risks include: prior caesarean delivery *n* = 878 (6.7%), prior neonatal death *n* = 549 (4.2%), chronic hypertension *n* = 357 (1.8%), and chronic diabetes *n* = 66 (0.3%) (Figure 1).

While women with high‐risk pregnancies experienced more adverse care processes compared to women with low‐risk pregnancies, low‐risk pregnancies made up 43%–55% of the total adverse delivery process outcomes (Table 2). Unplanned c‐sections occurred for 891 (10.7%) of high‐risk pregnancies (95% CI: 9.5–12.1) compared to 692 (5.6%) of low‐risk pregnancies (95% CI: 5.0–6.2). In total, of the 1583 women who had unplanned c‐sections, 692 (43.7%) were among women with pregnancies classified as low risk. Similarly, women who reported concerns about delivery complications experienced more unplanned c‐sections than women reporting no concerns about complications: *n* = 255 (9.3%, 95% CI: 7.8–11.0) versus. *n* = 1278 (7.3%, 95% CI: 6.6–7.9). Among the 1583 women who experienced unplanned c‐sections, 1278 (80.7%) were among women who had reported no concerns about delivery complications at study enrollment.

**TABLE 2:** Intermediate outcomes report by pregnancy risk assessments and self‐reported concerns about complications.

	Unplanned c‐section		Intrapartum referral		Blood transfusion		Hysterectomy		ICU admission	
	*N* (%) [95% confidence interval]	*p*‐value	*N* (%) [95% confidence interval]	*p*‐value	*N* (%) [95% confidence interval]	*p*‐value	*N* (%) [95% confidence interval]	*p*‐value	*N* (%) [95% confidence interval]	*p*‐value
All pregnancies	1583 (7.52) [6.92–8.17]	‐	1771 (8.92) [8.14–9.76]	‐	334 (1.64) [1.36–1.96]	‐	63 (0.32) [0.22–0.47]	‐	42 (0.20) [0.14–0.30]	‐
Provider assessment										
High‐risk pregnancies	891 (10.71) [9.49–12.08]	0.00	804 (10.38) [9.27–11.60]	0.00	178 (2.42) [2.00–2.94]	0.00	36 (0.44) [0.25–0.75]	0.00	26 (0.31) [0.18–0.55]	0.03
Low‐risk pregnancies	692 (5.57) [5.04–6.16]		967 (8.02) [7.18–8.96]		156 (1.15) [0.85–1.58]		27 (0.25) [0.15–0.42]		16 (0.14) [0.07–0.28]	
Percent from low‐risk pregnancies	43.71%		54.60%		46.71%		42.86%		38.10%	
Self‐reported concerns about complications										
Somewhat/moderately/extremely concerned about complications	255 (9.31) [7.83–11.03]	0.00	281 (11.01) [9.44–12.79]	0.00	44 (1.64) [1.09–2.46]	0.68	10 (0.23) [0.11–0.49]	0.52	7 (0.32) [0.14–0.71]	0.31
Not at all/slightly concerned about complications	1278 (7.24) [6.61–7.93]		1438 (8.59) [7.81–9.44]		277 (1.64) [1.33–2.01]		49 (0.32) [0.20–0.50]		32 (0.17) [0.11–0.27]	
Percent from women reporting not at all/slightly concerns	80.73%		81.20%		82.93%		77.78%		76.19%	

Intrapartum referrals occurred among 1771 (8.9%) of women in the total sample. Women with high‐risk pregnancies experienced more intrapartum referrals compared to women with low‐risk pregnancies: *n* = 804 (10.4%, 95% CI: 9. 3–11. 6) versus *n* = 967 (8.0%, 95% CI: 7.2–9.0). However, among the 1771 women who experienced a referral during delivery, 967 (54.6%) were among women with pregnancies classified as ‘low‐risk’. Women who reported concerns about delivery complications also experienced higher rates of intrapartum referrals compared to women reporting no concerns: 281 (11.0%, 95% CI: 9. 4–12.8) versus 1438 (8.6%, 95% CI: 7.8–9.4). Among all women who experienced an intrapartum referral, 1438 (81.2%) reported having no concerns about delivery complications.

Blood transfusions occurred in 334 (1.6%) of the sample, with a higher frequency occurring among high‐ versus low‐risk pregnancies: 178 (2.4%, 95% CI: 2.0–2.9) versus 156 (1.2%, 95% CI: 0.85–1.58). Among the 334 women who had a blood transfusion, 156 (46.7%) were among low‐risk pregnancies. Women who reported concerns about complications had the same rate of blood transfusions as those without concerns: 44 (1.6%, 95% CI: 1.1–2.4) versus 277 (1.6%, 95% CI: 1.3–2.0). 82.9% of blood transfusions occurred among women without concerns of delivery complications (*n* = 277).

A total of 63 women (0.3% of the sample) reported having a hysterectomy during or immediately after delivery, which is indicated when post‐partum haemorrhage cannot be controlled through other means. This occurred among a higher percentage of women with high‐risk pregnancies compared to those with low‐risk pregnancies: 36 (0.4%, 95% CI: 0.3–0.8) versus 27 (0.3%, 95% CI: 0.2–0.4). Twenty‐seven (42.9%) of the hysterectomies occurred among low‐risk pregnancies. Among women reporting concerns about delivery complications, 10 (0.2%, 95% CI: 0.1–0.5) had a hysterectomy, and among women reporting no concerns, 49 (0.3%, 95% CI: 0.2–0.5) had a hysterectomy. Among all women who reported a hysterectomy during delivery, 49 (77.8%) were among women who previously reported no concerns about delivery complications.

ICU admissions occurred among 42 women (0.2% of the sample) and ICU admissions were higher among high‐risk compared to low‐risk pregnancies: 26 (0.3% 95% CI: 0.2–0. 6) versus 16 (0.1%, 95% CI: 0.1–0.3). Sixteen (38.1%) of ICU admissions were for low‐risk pregnancies. Among women reporting concerns about delivery complications, 7 (0.3%, 95% CI: 0.1–0.7) experienced an ICU admission, compared to 32 (0.1%, 95% CI: 0. 1–0. 3) women who reported no concerns about complications. Among the 42 women who were admitted to the ICU, 32 (76.2%) were women reporting no concerns about delivery complications.

Mortality was more frequent among pregnancies classified as high‐risk (Table 3). However, within each mortality category, 53% of mortality was among low‐risk pregnancies.

**TABLE 3:** Adverse delivery outcomes report by pregnancy risk assessments and self‐reported concerns about complications.

Outcome	Stillbirth		Neonatal death		Maternal death		Composite mortality	
	*N* (%) [95% confidence interval]	*p*‐value	*N* (%) [95% confidence interval]	*p*‐value	*N* (%) [95% confidence interval]	*p*‐value	*N* (%) [95% confidence interval]	*p*‐value
All pregnancies	83 (0.44) [0.32–0.60]	‐	359 (1.90) [1.67–2.15]	‐	31 (0.18) [0.10–0.32]	‐	407 (2.13) [1.89–2.41]	‐
Provider assessment								
High‐risk pregnancies	38 (0.52) [0.35–0.79]	0.19	162 (2.20) [1.74–2.79]	0.01	11 (0.19) [0.09–0.40]	0.71	184 (2.54) [2.02–3.18]	0.01
Low‐risk pregnancies	45 (0.39) [0.26–0.57]		197 (1.71) [1.48–1.98]		20 (0.17) [0.09–0.32]		223 (1.89) [1.64–2.18]	
Percent from low‐risk pregnancies	54.22%		54.87%		64.52%		54.79%	
Self‐reported concerns about complications								
Somewhat/moderately/extremely concerned about complications	13 (0.58) [0.31–1.09]	0.48	56 (2.00) [1.40–2.87]	0.09	7 (0.44) [0.12–1.69]	0.24	64 (2.34) [1.69–3.22]	0.06
Not at all/slightly concerned about complications	66 (0.42) [0.29–0.61]		293 (1.86) [1.63–2.12]		24 (0.14) [0.08–0.25]		331 (2.08) [1.82–2.38]	
Percent from women reporting not at all/slightly concerns	79.52%		81.62%		77.42%		81.33%	

Stillbirths occurred among 38 pregnancies that were classified as high‐risk (0.5%, 95% CI: 0.4–0.8) and among 45 pregnancies classified as low‐risk (0.4%, 95% CI: 0.3–0.6). Among the 83 stillbirths that occurred among the sample, 45 (54.2%) were among pregnancies classified as low‐risk. There were more stillbirths among women with concerns than those without concerns about delivery complications: 13 (0.6%, 95% CI: 0.3–1.1) versus 66 (0.4% 95% CI: 0.3–0.6), with 66 (80.0%) of stillbirths occurring in women reporting no concerns.

Neonatal deaths were reported by 359 women (1.9% of the sample). Rates of neonatal deaths also differed between high‐risk pregnancies *n* = 162 (2.2%, 95% CI: 1.7–2.8) and low‐risk pregnancies *n* = 197 (1.7%, 95% CI: 1.5–2.0). However, the 197 neonatal deaths that occurred in low‐risk pregnancies accounted for 55.0% of all neonatal deaths reported. Stratified by self‐reported concerns about complications, 56 (12.0%, 95% CI: 1.4–2.9) neonatal deaths occurred among women reporting concerns and 293 (1.9%, 95% CI: 1.6–2.1) among women reporting no concerns. The 293 neonatal deaths among women reporting no concerns about complications made up 81.6% of neonatal deaths in the sample.

Maternal death occurred among 31 women (0.2% of the sample). Maternal deaths occurred among 11 (0.2%, 95% CI: 0.1–0.4) high‐risk pregnancies versus 20 (0.2%, 95% CI: 0.1–0.3) among low‐risk pregnancies, with 20 (64.42%) maternal deaths occurring in pregnancies classified as low‐risk. Seven (0.5%, 95% CI: 0.1–0.1.7) of maternal deaths occurred among women reporting concerns about delivery complications versus 24 (0.1%, 95% CI: 0.1–0.3) among women without concerns, with these 24 deaths accounting for 77.4% of all maternal deaths in the sample.

Four hundred and seven (2.1%) of pregnancies experienced any mortality event as assessed by a measure of composite mortality. Mortality events occurred among 184 (2.5%, 95% CI: 2.0–3.2) pregnancies classified as high‐risk and among 223 (1.9%, 95% CI: 1.6–2.2) pregnancies classified as low‐risk. The 223 mortality events among low‐risk pregnancies accounted for 55.0% of mortality in the sample. Among women reporting concerns about delivery complications, 64 (2.3%, 95% CI: 1.7–3.2) experienced a mortality event compared to 331 (2.1%, 95% CI: 1.8–2.4) of women reporting no concerns. Of the 407 mortality events in the sample, these 331 composite mortality cases that occurred among women reporting no concerns of delivery complications accounted for 81.3% of all mortality in the sample.

An intervening variable which could moderate the effect of pregnancy risk on adverse outcomes is place of delivery. Therefore, in supplementary analyses, we analysed place of delivery by assessed level of pregnancy risk, comparing high and low risk pregnancies as well as whether deliveries happened at primary health care, hospital, or referral hospital level. There was no detectable difference in percentage of mothers with high risk versus low risk pregnancies who gave birth at home, in primary health centres, or in a set of designated Level Four “delivery hub” hospitals. High risk deliveries were however more likely to take place at the highest referral level, Kakamega County General Hospital (5.8% high risk pregnancies versus 4.2% of low risk pregnancies, *p* = 0.00).

## DISCUSSION

### Main findings

In this analysis, we find that women who fulfil standard clinical criteria for pregnancy risk or self‐identify as high‐risk have somewhat higher incidence of delivery complications (intrapartum referral, unplanned c‐sections, and maternal complications) compared to those whose pregnancies are classed as low risk. We also find higher rates of adverse delivery outcomes, including neonatal and maternal deaths, among pregnancies classified as high‐risk compared to low‐risk pregnancies. While it is unsurprising to see that measured risks are correlated with adverse outcomes, the goal of identifying risks is not to identify correlations but to improve population health outcomes, which requires that measures have robust predictive power. Here the predictive performance of risk measures is less impressive: it failed to identify most mothers who experienced adverse pregnancy outcomes. Specifically, women with pregnancies classified as low‐risk account for the majority of the population experiencing these adverse outcomes: notably, pregnancies without any known risks for delivery complications comprise over 54% of all mortality in our sample.

These findings in Kakamega County may reflect broader trends in Kenya, as the rates of adverse outcomes are comparable to recent findings from national surveys in Kenya, notably the DHS 2022 which reported a national neonatal mortality rate of 21 deaths per 1000 live births, and a neonatal mortality rate of 16 per 1000 births for Kakamega County. The mortality rate among all pregnancies in this sample is comparable to these results from national surveys.

Our results are consistent with recent evidence from other low‐ and middle‐income settings, which find that most adverse newborn outcomes take place among women identified as low‐risk by national guidelines. These findings are also consistent with other analyses from western Kenya, which find high rates of complication during pregnancy and delivery among those rated as both low and high‐risk at ANC [11]. Indeed, despite the widespread de facto reliance on risk stratification in low‐ and middle‐income settings, emerging evidence suggests that this model merits re‐examination for decision making at the individual level. For example, a recent study from the United States suggests that, while high‐risk status is predictive of unexpected complications, this was not a sensitive indicator: almost one third of pregnancies labelled low‐risk experienced an unexpected complication [5]. Resource‐limited settings face more challenges given lower ANC coverage and fewer diagnostic tests during ANC. For example, analysis of India's 2019–2021 National Family Health Survey demonstrated that 47% of newborn deaths and 56% of stillbirths take place among pregnant women classified as low‐risk by national guidelines [3]. A 2018 systematic review of risk prediction models found that even the best models were not accurate enough to be used for clinical decision‐making [4], while a Cochrane Review of risk stratification to prevent preterm birth found that the effect of risk scoring systems was unknown due to lack of adequate evidence [1]. These findings suggest that existing measures of risk stratification in both high‐income and low‐income settings overlook the potential that ‘low‐risk’ pregnancies can experience life‐threatening complications during delivery. Our findings advance this literature by evaluating how effectively pregnancy‐risk classifications predict childbirth complications in a setting representative of many regions with high maternal and neonatal mortality.

Our findings also have implications for Kenya's maternal and child health strategies. Kenya has implemented a range of strategies to prevent maternal and newborn deaths in recent years, including strengthening antenatal care (ANC) and making deliveries free of charge. These policies have succeeded in increasing the rates of antenatal care utilisation and facility‐based childbirth [6]. However, progress in reducing mortality has slowed over this period—for example, newborn mortality did not decline between the 2014 and 2022 Demographic and Health Surveys.

One approach to addressing this is by improving risk screening, including several changes captured in the recently updated National Obstetrics Guidelines, such as increased point of care ultrasound, improved anaemia screening, and other ANC quality improvements. However, while improved screening is certainly warranted, this paper's findings suggest that risk prediction has a long way to go. As evidence from high‐income countries shows, a significant fraction of serious complications will likely remain unanticipated even as screening improves. Another approach is to strengthen the quality of intrapartum care in lower‐level facilities: approximately 1/3 of women in Kakamega deliver in lower‐level facilities that lack the ability to manage obstetric emergencies [12]. However, several recent studies have found that intrapartum quality improvement interventions have not reduced mortality when implemented in settings without advanced services such as Caesarean section, blood transfusion, and advanced newborn care [13, 14, 15].

A different approach, known as Service Delivery Redesign for Maternal and Newborn health, was proposed by the Lancet Commission on High Quality Health Systems [16]. These reforms would reorganise antenatal and delivery care to ensure that all women give birth in facilities equipped to handle life‐threatening emergencies. This approach is motivated in part by the recognition that pregnancy risk cannot be easily predicted and so relies on having deliveries take place, as much as possible, in care settings which can provide definitive care for complications. Prospective evaluation of SDR and other strategies to address gaps in risk stratification is therefore warranted to build the evidence base and address gaps in current care models.

### Strengths and limitations

Our study has several strengths. First, we use a large population‐based sample in a rural, low‐income setting. This is prospective data, collected from women who were enrolled before delivery and re‐interviewed at two time points shortly after delivery. By contrast, the emerging literature on adverse outcomes among low‐risk pregnancies largely relies on retrospective data from pregnancies several years in the past [2, 3]. Our prospective study design likely reduces recall bias, enhancing the accuracy of reported risk factors and delivery experiences. Second, our analysis includes care process outcomes, including intrapartum referrals, unplanned c‐sections, and maternal near‐miss events. Including these outcomes helps shed light on events during deliveries that may contribute to mortality outcomes.

Our primary data is limited to self‐reported pregnancy risks and outcomes. While self‐reported risks are not equivalent to using clinical information from medical records to measure risk, this self‐reported knowledge of risk has a distinct advantage, in that it reflects women's own understanding of their own pregnancy risks. This subjective understanding is highly relevant to care‐seeking decisions made by women and their families. Another limitation is that we rely on women's self‐reported account of their delivery. Nonetheless, self‐reported recall of delivery process outcomes has been validated in studies of severe maternal morbidity [10]. Moreover, we conducted periodic data validation exercises throughout the survey period, in which we revisited study facilities to obtain facility records for all reported mortality events.

### Interpretations

Identification of higher risk pregnancies is a key function of antenatal care in every setting. However, in high‐income settings, identification of risk during antenatal care triggers additional specialist attention, diagnosis, and treatment, and potentially selection of a more specialised birth facility—in contexts where virtually all women give birth in designated, well‐resourced hospitals. By contrast, in lower‐resource settings, risk stratification has been used in practice to counsel women with “low‐risk” pregnancies to seek care in lower‐level care settings, while those designated as high‐risk are often advised to give birth in higher‐level facilities such as district or regional hospitals. This could be an effective practice if measures of risk were highly sensitive, if lower‐level facilities were able to consistently provide adequate care for life‐threatening complications, or if emergency referral systems were extremely fast and reliable. However, in many settings with elevated maternal and newborn mortality, several of these assumptions are often not valid. In this paper, we demonstrate the limited sensitivity of existing measures of risk.

## CONCLUSION

At current rates of progress, many low and middle‐income countries are unlikely to achieve the Sustainable Development Goal targets for reduction of maternal and newborn mortality. A key challenge in this effort is making sure that all women and newborns who experience life‐threatening complications during childbirth receive timely, high‐quality care. The findings presented in this paper make clear that the current practice in many low‐ and middle‐income settings fails to identify truly high‐risk pregnancies, with negative consequences for the health of mothers and newborns [17, 18].

## FUNDING INFORMATION

Funding was provided by the Bill and Melinda Gates Foundation grant number: INV‐028724.

## Supporting information


**FIGURE S1.** Schema of survey time points.
**Table S1:** Delivery locations.
