# Supplementary material for: Risk stratification during antenatal care failed to identify most mothers who experienced adverse pregnancy outcomes: A prospective study from Kakamega County, Kenya
Source: Trop Med Int Health. 2025 Apr 6;30(6):531–8. doi: 10.1111/tmi.14110 (PMC12136940; doi:10.1111/tmi.14110)

**Supporting information:** Supplemental table 1. Delivery locations


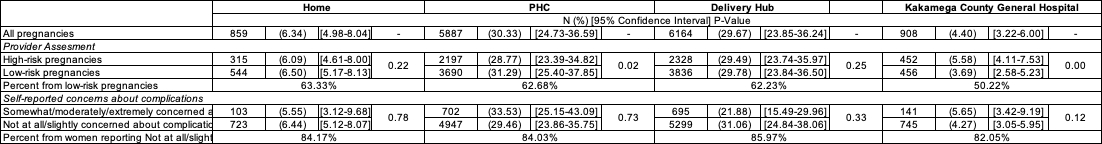


**Supporting information** Supplementary Figure 1. Schema of survey time points


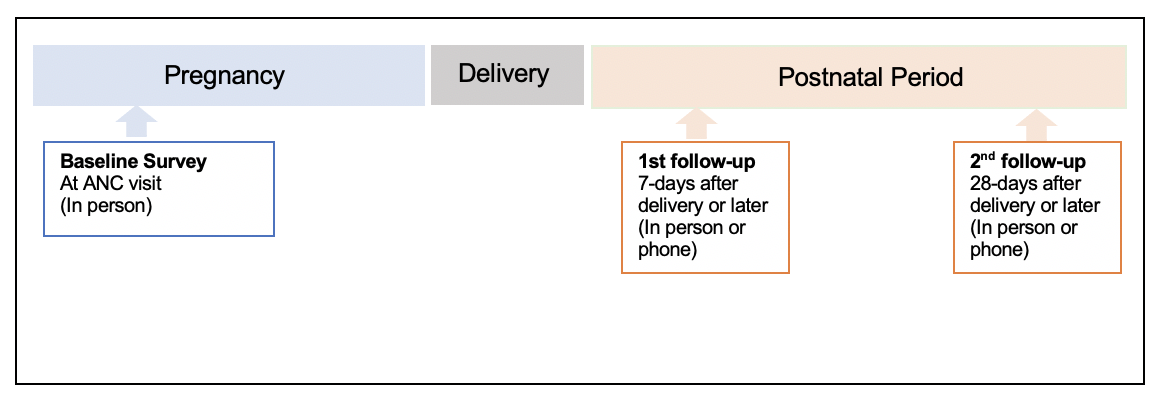

Supplement: Supplementary file 1 — FIGURE S1. Schema of survey time points. Table S1: Delivery locations. [file TMI-30-531-s001.docx]
